# Covariate Model of Pixel Vector Intensities of Invasive *H. sosnowskyi* Plants

**DOI:** 10.3390/jimaging7030045

**Published:** 2021-03-03

**Authors:** Ignas Daugela, Jurate Suziedelyte Visockiene, Egle Tumeliene, Jonas Skeivalas, Maris Kalinka

**Affiliations:** 1 Department of Geodesy and Cadastre, Vilnius Gediminas Technical University, Sauletekio av. 11, LT-10223 Vilnius, Lithuania; ignas.daugela@vilniustech.lt (I.D.); egle.tumeliene@vilniustech.lt (E.T.); jonas.skeivalas@vilniustech.lt (J.S.); 2 Antanas Gustaitis’ Aviation Institute, Vilnius Gediminas Technical University, Sauletekio av. 11, LT-10223 Vilnius, Lithuania; 3 Institute of Land Management and Geomatics, Vytautas Magnus University Agriculture Academy, Studentu 11, Akademija, LT-53361 Kaunas District, Lithuania; 4 Department of Geomatics, Riga Technical University, 1 Kalku Street, LV-1658 Riga, Latvia; Maris.Kalinka@rtu.lv

**Keywords:** *H. sosnowskyi*, colour intensity, covariance function, quantisation interval, correlation coefficient

## Abstract

This article describes an agricultural application of remote sensing methods. The idea is to aid in eradicating an invasive plant called Sosnowskyi borscht (*H. sosnowskyi*). These plants contain strong allergens and can induce burning skin pain, and may displace native plant species by overshadowing them, meaning that even solitary individuals must be controlled or destroyed in order to prevent damage to unused rural land and other neighbouring land of various types (mostly violated forest or housing areas). We describe several methods for detecting *H. sosnowskyi* plants from Sentinel-2A images, and verify our results. The workflow is based on recently improved technologies, which are used to pinpoint exact locations (small areas) of plants, allowing them to be found more efficiently than by visual inspection on foot or by car. The results are in the form of images that can be classified by several methods, and estimates of the cross-covariance or single-vector auto-covariance functions of the contaminant parameters are calculated from random functions composed of plant pixel vector data arrays. The correlation of the pixel vectors for *H. sosnowskyi* images depends on the density of the chlorophyll content in the plants. Estimates of the covariance functions were computed by varying the quantisation interval on a certain time scale and using a computer programme based on MATLAB. The correlation between the pixels of the *H. sosnowskyi* plants and other plants was found, possibly because their structures have sufficiently unique spectral signatures (pixel values) in raster images. *H. sosnowskyi* can be identified and confirmed using a combination of two classification methods (using supervised and unsupervised approaches). The reliability of this combined method was verified by applying the theory of covariance function, and the results showed that *H. sosnowskyi* plants had a higher correlation coefficient. This can be used to improve the results in order to get rid of plants in particular areas. Further experiments will be carried out to confirm these results based on in situ fieldwork, and to calculate the efficiency of our method.

## 1. Introduction

Sosnowskyi borscht (*Heracleum sosnowskyi*) plants were widespread only in the Caucasus region before they were cultivated in the Soviet Union as a silage crop. As a result, the plant has spread to Russia, Ukraine, Belarus and the Baltic States as an invasive species. All parts of the plant accumulate a particularly strong allergen called furanocoumarin, and the juice contains substances that sensitise the skin to the sun and can cause first- to third-degree skin burns. *H. sosnowskyi* displace native plant species by overshadowing them, and are difficult to eradicate from rivers since floods carry the seeds. They spread very fast, at a rate of 60 m per year along river banks and roadsides, in large inflorescences. Each plant matures tens of thousands of seeds every year, and the largest may produce up to 100,000 seeds and scatter them within a radius of about 4 m. Up to 95% of the seeds can remain viable for several years. *H. sosnowskyi* is also difficult to eradicate because it sprouts from the roots, even after being cut down several times.

As technologies advance, remote sensing methods have improved through the use of smaller or more capable equipment, or by applying advanced processing and computational techniques, which in both cases gives more accurate results. The use of remote sensing techniques is therefore increasing rapidly, and has found new fields of application [1]. When remote sensing data are processed differently (via different methods), the results can save time, provide solutions to various social challenges, or may be more effective in terms of cost. When it comes to data analysis, i.e., identifying patterns and objects from the images, a human interpreter is superior to a computer, but the main disadvantage of a human interpreter is the relative subjectivity, which is impossible to avoid. While satellite images can be used alone to solve many problems, computer-assisted classification results (thematic maps) are good examples of data in raster format that are suitable for storage in a GIS [1]. Various tendencies can then be identified and ideas can be checked by calculating the correlations. Although correlation analysis has been available to researchers in theory for roughly 85 years, the availability of programming languages and computer programmes that allow it to be implemented became widely accessible only recently. The technique is an old one, but improvements are still emerging [2]. In the area of earth science, image correlation can be used to analyse and monitor any Earth’s surface displacements in the geosciences [3,4,5]. The method was developed and first applied to imaging processes in the early 1990s, and relies on a discrete Fourier transform [3]. In our work, the correlation coefficient depends on the distributions of the variables that are analysed and the forms of relationships that are evaluated. Data (images) from remote sensing form the basis for an analysis of the geometric characteristics of plants. The remote sensing methods of image segmentation and mapping are used to classify the pixels [6,7,8,9,10,11,12,13,14]. Imaging is an important method used in this research to assess the danger of invasive *H. sosnowskyi* plants. The unique spectral signatures (pixel values) of *H. sosnowskyi* have been observed to be much higher than those of neighbouring vegetation in the early crop growth stage (May) [15]. These differences in colour improve the chances of invasive plant detection and increase the likelihood of being able to monitor the invasion over time using multiple images [9]. Changes in the pixel values of satellite images can be seen, and the values of these pixels depend on the density of the chlorophyll content in plants and the reflectance spectra of all types of plant vegetation [16]. Another important factor is the spatial resolution of the pixels, which is referred to as the pixel size. This is the characteristic that determines the size of the objects that we can sense in the image [1]. *H. Sosnowskyi* plants can cover areas of 3 to 20 m^2^ or more (and the leaves can reach up to 3 m in length) [17]. Scientists have used multi-spectral Sentinel-2 high resolution imaging in four spectral bands for the assessment of small areas of vegetation [7,18,19]. Sentinel-2 imaging has a spatial resolution of 10 m and the bands are B2 Blue (490 nm), B3 Green (560 nm), B4 Red (665 nm) and B8 Narrow NIR (842 nm). Image pixels with a B4-B3-B2 composition (natural colour results) can be classified using methods such as a per-pixel support vector machine (SVM) (supervised) and per-field object-oriented (unsupervised) techniques [7,19].

In our work, we present data preparation methods and applications for the calculation of the correlation coefficients of pixels. For this task, we use a combination of two-pixel classification methods. The results of supervised classification of the image pixels classification are the spectral characteristics of the plants, or in other words their spectral signatures. This information is necessary to build a raster model of the *H. sosnowskyi* plants using an unsupervised classification method.

The correlation functions represent the values of the auto-covariance function for a given class of plants (in this case, *H. sosnowskyi* plants, where the other plants consist of agricultural vegetation) and a pixel or inter-covariance function for pairs of classes (for example for *H. sosnowskyi* and other plants) within the corresponding ranges of the colour wavelengths (quantisation intervals). By applying this theory, we determine the strength of the interdependency between the pixels representing the class of *H. sosnowskyi* plants and other plants on agricultural land. The changes in correlation for each class over a given time scale were determined as a function of the change in the time intervals with a change in quantisation interval.

The purpose of this analysis was to evaluate whether the values of the auto-covariance function coefficient for *H. sosnowskyi* plant pixel vectors differed from those of other plants and agricultural land in the images. In other words, this correlation can be used to determine whether there is a relationship between the cells of invasive plants and other plants or agricultural land. The objective of this study was to develop a method based on multi-spectral optical image segmentation (supervised and unsupervised) and to check the reliability of this method using correlation analysis. This image processing method has also been previously used to identify differences between gases in the atmosphere.

## 2. Materials and Methods

### 2.1. Field Study

**Figure 1:**
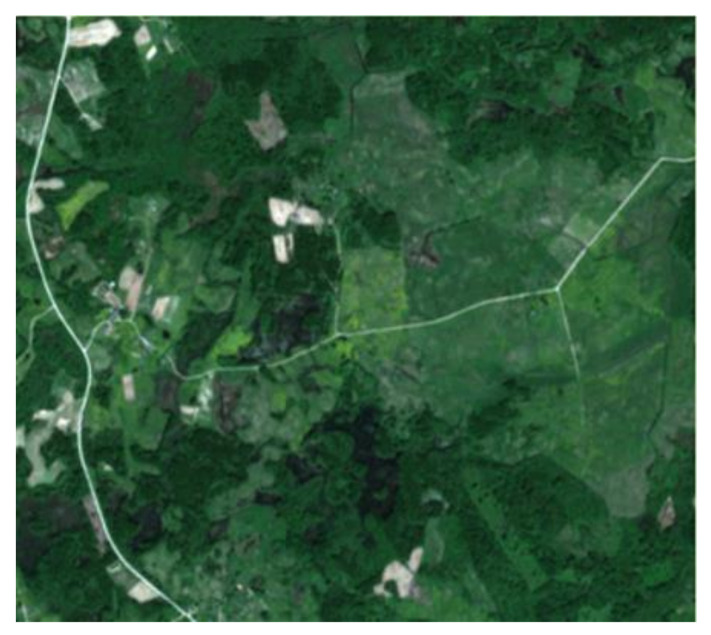
Sentinel-2A image taken on 10 April 2018 (B4-B3-B2 colour composite).

The district municipality of Vilnius is located in southeast Lithuania. The selected study area is agricultural, and has x-coordinates of between 6,099,369.02 m and 6,095,672.46 m and y-coordinates of between 395255.92 m and 401763.41 m in the Universal Transverse Mercator (UTM 35U zone) geographic coordinate system for the year 1984. The total area is 24 km^2^ (Figure 1). The research object is the population of invasive *H. Sosnowskyi* plants, which constitute one of the main threats to biodiversity. The chemical compounds found in invasive plants, including Sosnowskyi’s hogweed (*H. sosnowskyi*), are harmful to human health, affect soil and alter the overall ecosystem of the area. *H. sosnowskyi* are rich in essential oils that contain mostly aliphatic esters, with the organic compound octal acetate or octyl ethanoate (molecular formula C_10_H_20_O_2_) as the main constituent [20]. The substances present in these essential oils have strong, biologically active potential [20]. Although throughout Europe’s history, people have believed that the primary hazardous components of hogweeds were various coumarins (a vanilla-scented compound) [21,22,23], an article by Jakubska-Busse et al. (2013) investigated *H. sosnowskyi* and *Heracleum mantegazzianum* and showed that they contain many additional toxic components. *H. sosnowskyi* has coumarin derivatives that are the main cause of its photodynamic effect. The compounds contained in the essential oils of *H. sosnowskyi* and *H. mantegazzianum* may pose a risk to the eyes and cause irritation to the skin and respiratory system, dizziness, breathing difficulties, and nausea. It is possible that the observed effect of their activity is synergetic, i.e., a result of the combined activity of the two main groups of toxic compounds [22]. Controlling the distribution of invasive plant species requires a detailed knowledge of the composition, dynamics, habitats, environmental effects, pathways and ways in which each species population spreads.

This study used multi-spectral images from optical Earth observations by the Sentinel-2A satellite sensor (with a spatial resolution of 10 m), which were acquired on 10 April 2018 (Sentinel-2A, 2018). A colour composite (multi-spectral image) was constructed from Band 4 (red), Band 3 (green) and Band 2 (blue), and is a natural-coloured image. The invasive plant *H. sosnowskyi* has a light green colour and is clearly visible in the B4-B3-B2 spectral band.

### 2.2. Imaging Methodology

**Figure 2:**
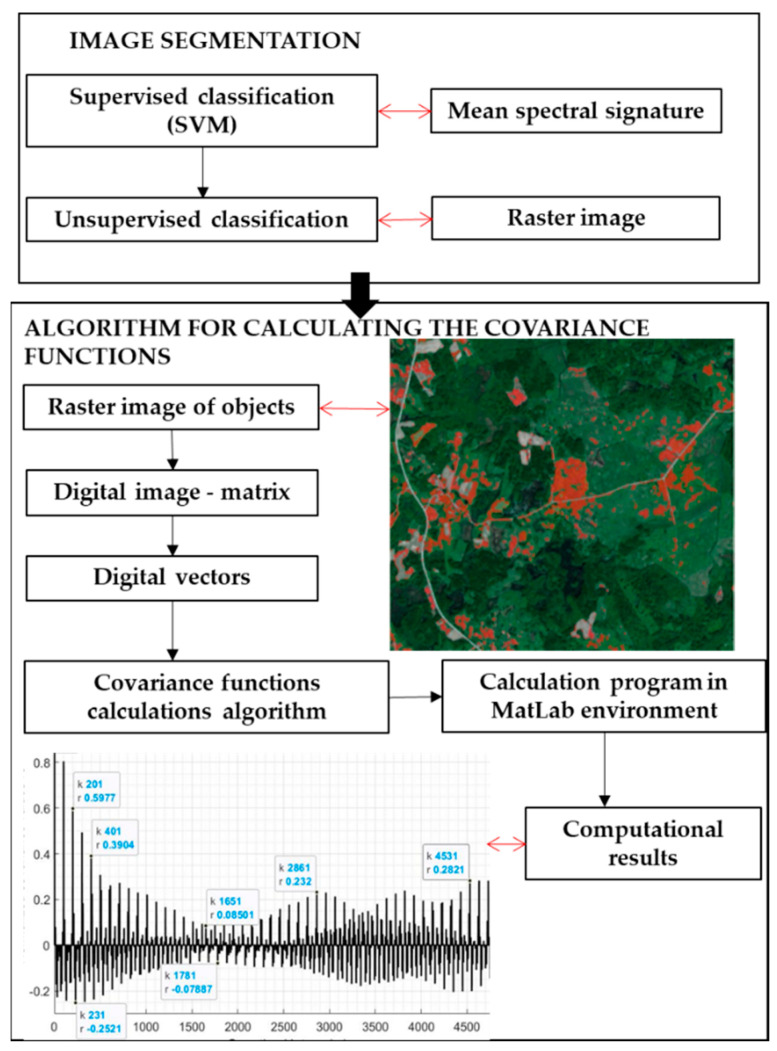
Schematic view of the image processing method used (source: authors).

This study was based on raster images of invasive *H. sosnowskyi* plants. In this case, satellite image classification was performed using two methods. The multi-spectral image pixels were classified using a combination of per-pixel SVM (supervised) and per-field object-oriented (unsupervised) classification methods based on the Sentinel-2A visible light bands (Figure 2) [8,12,15].

Supervised classification was performed based on manual interpretation. An operator digitalised the land cover boundaries using the GIS system (QGIS). A general description of the workflow is as follows: (i) the image data were downloaded using a semi-automatic classification plugin (SCP); (ii) these were automatically converted to surface reflectance; (iii) the work area was clipped; (iv) the ROIs were created by manually drawing a polygon or using an automatic region-growing algorithm; (v) a classification preview was created (the results of the land cover classification step) and was output; (vi) the quality of the classified lands was analysed.

Images such as those from Landsat or Sentinel are composed of several bands and a metadata file, which contains the information required for the conversion to reflectance. Images in the form of radiance can be converted to top of atmosphere (TOA) reflectance (combined surface and atmospheric reflectance) to reduce the between-scene variability, using a normalisation scale for solar irradiance. This TOA reflectance ($q_{p}$), which is a dimensionless ratio of the reflected energy to the total power [24], is calculated by:

*q_p_* = (π ∗ Lλ ∗ d^2^)/(ESUNλ ∗ cosθs)

where Lλ is the spectral radiance at the sensor’s aperture (at-satellite radiance); d is the Earth-Sun distance in astronomical units (provided in the Sentinel-2 metadata file); ESUNλ is the mean solar exo-atmospheric irradiance; and θs is the solar zenith angle in degrees, which is equal to θs = 90°-θe where θe is the Sun’s elevation.

It is worth pointing out that Sentinel-2 (Landsat 8) images are provided with band-specific rescaling factors that allow for the direct conversion from pixel digital numbers to TOA reflectance.

After supervised classification is complete, we obtain the mean spectral signature of *H. sosnowskyi* land cover. This is needed in order to classify the image pixels using an unsupervised (automatic) classification method. As part of an unsupervised classification approach, eCognition tools can be used to apply the ISODATA algorithm. ISODATA stands for “iterative self-organizing data analysis technique” and categorises continuous pixel data into classes/clusters with similar spectral-radiometric values. The output of an unsupervised classification process in eCognition is normally a raster image. The image result was transformed into a new form (a matrix of numbers) and was then analysed using a correlation model in MATLAB [25].

The pixel (*i*) column of matrix ($B_{i}$) can be understood as single-pixel column intensity vector. To process the images, a discrete transformation was applied to the known formula of mathematical statistics [26,27]. Estimates of the auto-covariance function of a geometrical characteristic of the plant, a single parametric vector of invasive plants, and the covariance functions of three numerical parametric vectors (φ) (representing *H. sosnowskyi*, other plants and agricultural land) were calculated by propagating data vectors in the form of random functions. The auto-covariance and inter-covariance functions of these functions were considered at various pixel quantisation intervals (*k*) [28]. The quantisation interval can be defined as the distance between the corresponding pixels of the image, for which the values can vary from one pixel to *n*/2 pixels, where *n* is the total number of pixels in the image.

When examining the entire surface of an image matrix, a variable covariance function was applied using the theory proposed in an article by Skeivalas and Parseliunas (2013) [28]. In this analysis, a covariate model of pixel vector intensities of invasive plants was calculated using the following four stages (Figure 2):

1. The image was transformed into a number matrix (array) $B_{i}$.
2. The numerical matrix was transformed into vectors.
3. Calculations were carried out using the covariance function algorithm in a MATLAB environment. The parameters of the covariance functions were described for *H. sosnowskyi*, other plants and agricultural land.
4. The correlation coefficient of the pixel vectors was calculated and the results were visualised.

Each column of an image pixel matrix can be understood as a single-pixel intensity vector. The array of column vectors of a matrix creates a transformed vector matrix of pixel intensity vectors of a transformed factor image, where *i* is the pixel intensity vector matrix for the image. The array of column vectors in the matrix creates a pixel intensity vector matrix $B_{i}$ for the transformed image *i*.

We obtain three matrices $B_{i}$ for the intensity parameters of three classes of plants: *H. sosnowskyi*, other plants and agriculture land, where *i* = 1, 2, 3. The column vectors of each matrix form the parametric vector of the whole matrix (Equation (1)):

(1) $$B_{1}=reshape\left(sm_{1r,}n_{1}{*m}_{1,1}\right)\;B_{2}=reshape\left(sm_{2g,}n_{1}{*m}_{1,1}\right)\;B_{3}=reshape\left(sm_{3b,}n_{1}{*m}_{1,1}\right)$$

where *reshape* is a MATLAB procedure (equation); $sm_{1r}$,$sm_{2g}$, $sm_{3b}$ are the image clips; and $n_{1}$, $m_{1,1}$ are the dimensions.

In the image, each class of pixel columns—vector (*φ*) classified by *H. sosnowskyi*, other plants and agriculture land—has the trend eliminated $\bar{\phi }$ (vector average) from the measurement data for that vector, so that systematic errors do not interfere with calculations. The random function based on the vector of plant pixels (*φ*) will be constant when its average is constant (MΔ=const→0), and the covariance function will depend only on the quantisation interval i.e., the difference in the arguments (*τ*). An algorithm for calculating the auto-covariance function for one random vector or the covariance function for two random vectors was introduced by Koch (2000), and is shown in Equations (2) and (3) [5]:

(2) $$K_{\phi }\mathrm{(\tau )}=M\left\{\delta {\bar{\phi }}_{1}\left(u\right)\cdot \delta {\bar{\phi }}_{2}\left(u+\tau \right)\right\},$$

or

(3) $$K_{\phi }\left(\tau \right)=\frac{1}{T-\tau }\int_{0}^{T-\tau }\delta \phi_{1}\left(u\right)\delta \phi_{2}\left(u+\tau \right)du,$$

From the available data for the calculation of the plants’ geometric characteristics coefficients used: where $\delta \phi_{1}=\phi_{1}-{\bar{\phi }}_{1}$, $\delta \phi_{2}=\phi_{2}-{\bar{\phi }}_{2}$ are the cantered *ϕ* vectors, where the trend is ${\bar{\phi }}_{}$, ***u*** is the parameter of the vectors, τ=k⋅Δ is the variable quantisation, k is the quantisation interval, *Δ* is the value of the unit, *M* is the symbol for the average; and *T* is time.

An estimate of the covariance function can be made based on the available data for the calculation of the geometric characteristic, as shown in Equation (4):

(4) $$K_{\phi }^{\prime }\left(\tau \right)=K_{\phi }^{\prime }\left(k\right)=\frac{1}{n-k}\sum_{i=1}^{n-k}\delta \phi_{1}\left(u_{i}\right)\delta \phi_{2}\left(u_{i+k}\right),$$

where *n* is the total number of pixels.

Equation (4) can be applied in the form of an auto-covariance or an inter-covariance function. When a function is auto-covariant, the vectors $\phi_{1}\left(u\right)$ and $\phi_{2}\left(u+\tau \right)$ form parts of a single vector, and when they are covariate, they are two different vectors.

The accuracy of the results and its significance are estimated using Equation (5):

(5) $$\sigma_{r}=\frac{1}{\sqrt{k}}\left(1-r^{2}\right)$$

where *r* is the correlation coefficient.

## 3. Experimental Results

The first part of this section describes the data results for an acquired raster image of *H. sosnowskyi* plants. Following this, we present the results of applying the correlation model in MATLAB and an analysis.

### 3.1. Image Segmentation Results

#### 3.1.1. Supervised Image Pixel Classification

**Figure 3:**
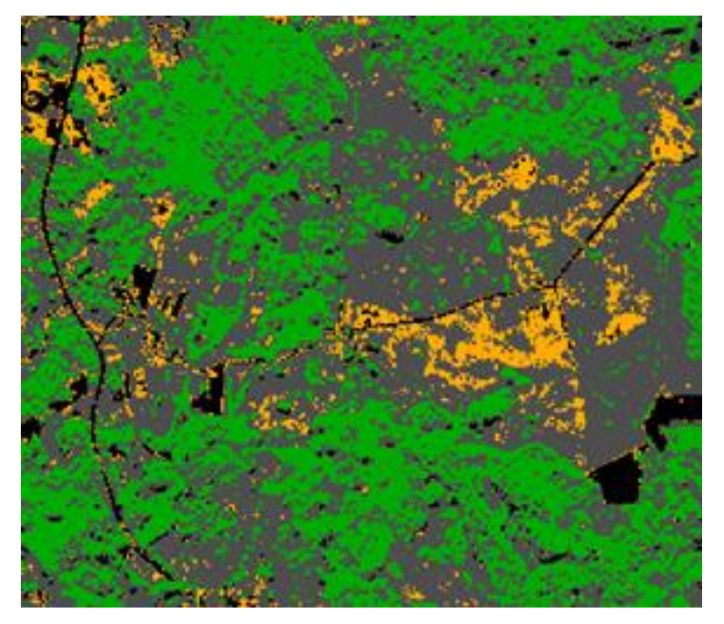
Image pixels classified by the support vector machine (SVM) method (minimum distance algorithm): *H. sosnowskyi*—yellow; forest—green; pathways—black (Source: authors).

In the test area, three crop classes were selected: forests, pathways and *H. sosnowskyi*. The multi-spectral image pixels were classified using QGIS software (Figure 3). The spectral characteristics of the classes were calculated based on the values of the pixels in the region of interest (ROI) using minimum distance algorithms.

**Figure 4:**
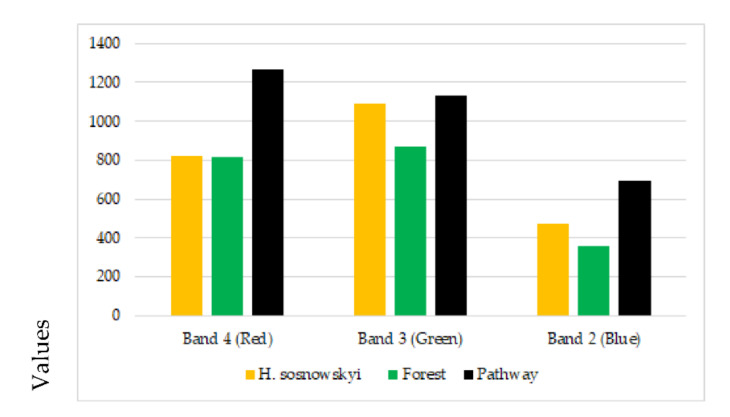
Results for crop classes based on spectral signature. A mean spectral signature of 956 for *H. sosnowskyi* was estimated based on supervised classification.

Classification results for the mean spectral signature for the selected crop classes are given in Table 1 and Figure 4.

The mean spectral signature for the *H. sosnowskyi* plants obtained in this study was 956, a higher value than that previously established for abandoned land (weeds or shrub) by Sužiedelytė Visockienė et al. (2020), who reported a value of 795 [12]. Areas of *H. sosnowskyi* growth are also classified as abandoned land, and hence to ensure full identification of abandoned land, we would recommend using mean spectral signature values of 800 to 1000 for segmentation via an unsupervised method.

The image pixel classification process was also used to calculate the differences in spectral characteristics between the spectral signatures of image pixels, based on the Euclidean distance, the spectral angle, and the Bray–Curtis similarity [11]. The traditional Euclidean distance involves a straightforward summation of the pixel-wise intensity differences, meaning that a small deformation may result in a large Euclidean distance (Table 2).

The values for the spectral angle of 8–12° show that all of the selected classes are relatively similar, but that the similarity between the *H. sosnowskyi* and forest classes is lower. Consequently, these two classes have different mean spectral signatures.

One method of assessing the image segmentation results is to calculate the kappa coefficient and overall accuracy, by comparing the pixel classification with undependable input for training. The kappa scale is from zero to one, and pixel classification is highly accurate when the kappa coefficient is close to one. The results were compared with those of the spectral angle mapping algorithm for pixel classification. The value of the kappa coefficient was 0.95, with an overall accuracy of 97.4%. The data are therefore suitable for use in the next calculation step, which involves segmentation based on the mean spectral signature.

#### 3.1.2. Image Segmentation Using Mean Spectral Signature

**Figure 5:**
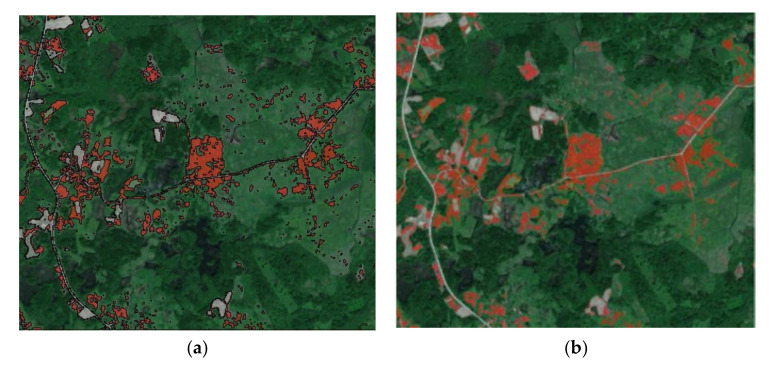
Raster images of *H. sosnowskyi* plants: (**a**) merging of results; (**b**) image used for correlation processes (*H. sosnowskyi*—red, other plants—green, agricultural land—grey).

Unsupervised image segmentation was done using the commercial software eCognition, using a method consisting of pixel classification based on a conceptual hierarchical network of classes and fuzzy logic for the combination and judgment of rules [11]. The stages in this work were as follows: (i) selection of the smallest segmentation area (five pixels); (ii) threshold segmentation/classification of the *H. sosnowskyi* crop class, based on a mean spectral signature of 950; (iii) object merging (as shown in Figure 5a); and (iv) smooth object smoothing (as shown in Figure 5b).

### 3.2. Results from the Correlation Function Model

#### 3.2.1. Differences in Covariance Values for Plant Classes

Values of the auto-covariance function were calculated for three classes of plants: *H. sosnowskyi* (kfr1), other plants (kfr2) and agricultural land (kfr3). The inter-covariance function was calculated between pairs of plants: *H. sosnowskyi* and other plants (kfr12); other plants and agricultural land (kfr23); and *H. sosnowskyi* and agricultural land (kfr13). The respective quantisation intervals *k* were from zero to 5000.

**Figure 6:**
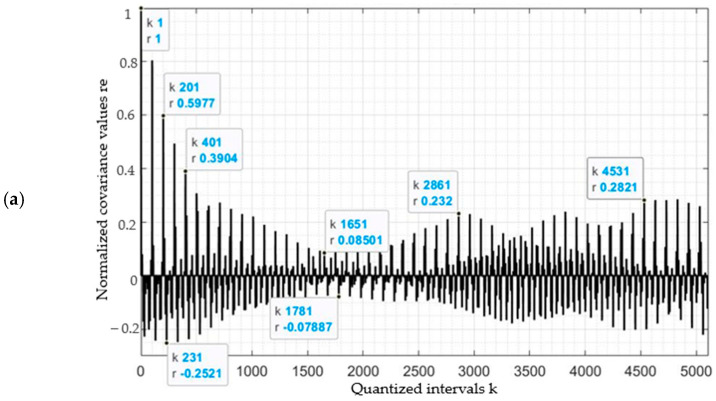

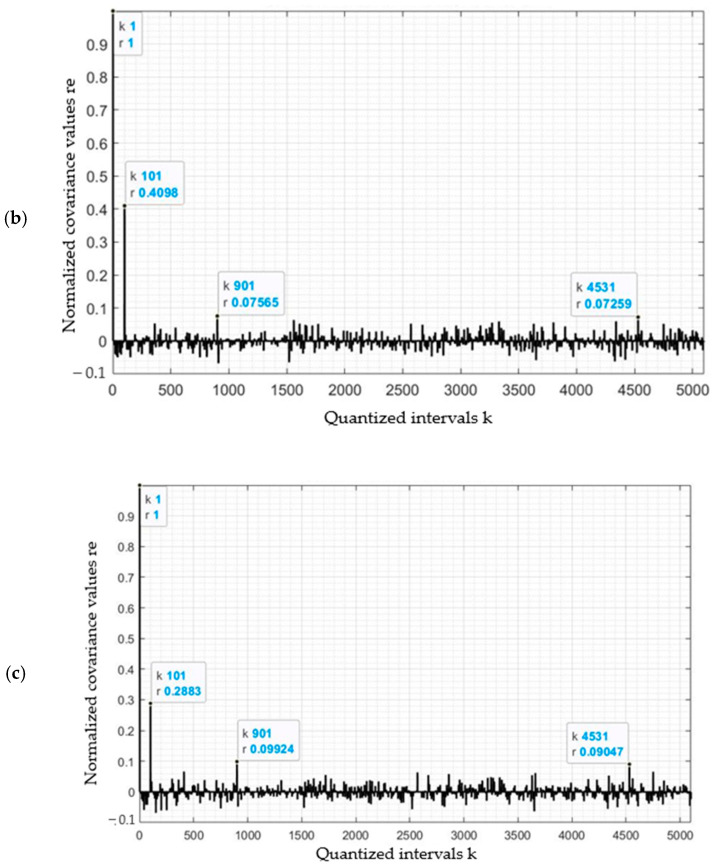
Pixel vector auto-covariance functions: (**a**) kfr1—*H. sosnowskyi* plants (shown in red in Figure 5); (**b**) kfr2—other plants (shown in green in Figure 5); (**c**) kfr3—agricultural land (shown in grey in Figure 5).

**Figure 7:**
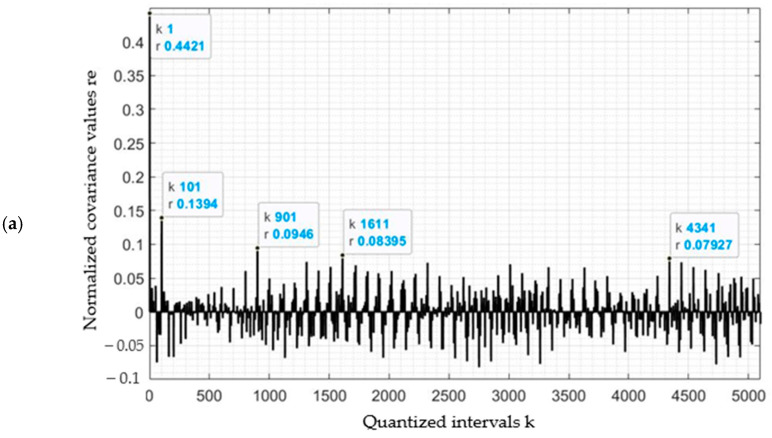

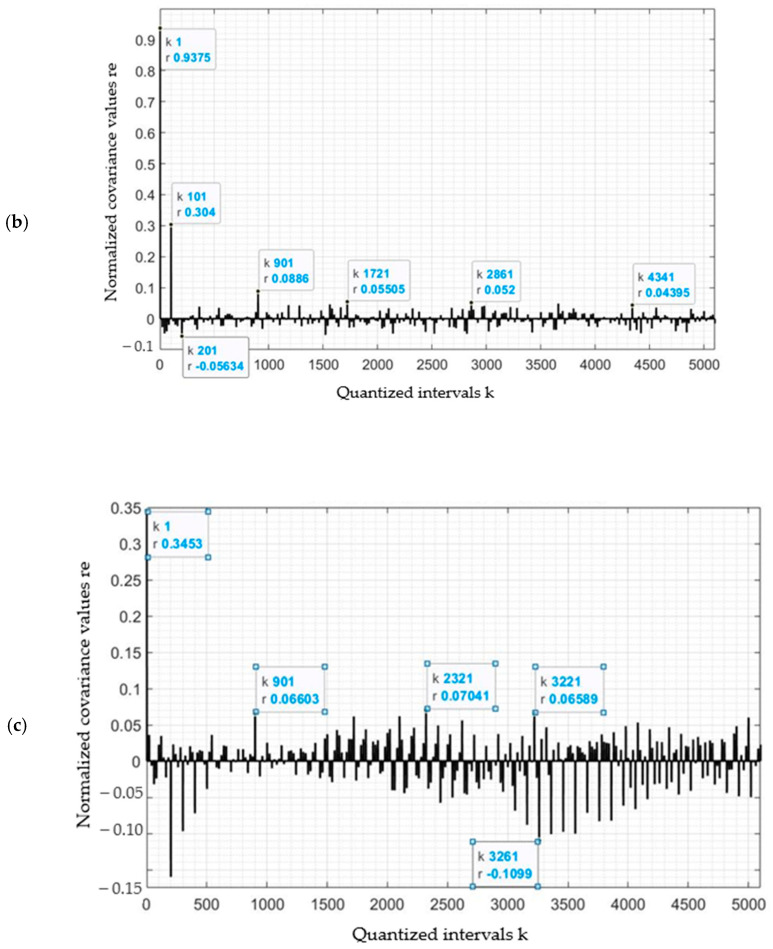
Covariance between pixel vectors: (**a**) kfr12–*H. sosnowskyi* plants (shown in red in Figure 5) and other plants (shown in green in Figure 5); (**b**) kfr23—other plants (shown in green in Figure 5) and agricultural land (shown in grey in Figure 5); (**c**) kfr13—*H. sosnowskyi* plants (shown in red in Figure 5) and agricultural land (shown in grey in Figure 5).

The covariance values for the kfr1, kfr2 and kfr3 plant classes showed differences between the geometric characteristics during the spring growth period. The total number of pixels in the raster image was *n* = 10,000, and the values used to calculate the quantisation interval (*k*) therefore ranged from zero to 5000. In the following figures, the quantisation interval (*k*) is shown on the abscissa, and the values of the correlation coefficients (*r*) are shown on the ordinate (Figure 6 and Figure 7).

The covariance values for the auto-covariance function (*r*) for *H. sosnowskyi* (kfr1) plants were distinct from the other plants (Figure 6a). The highest correlation coefficient value was r→1.0 for quantisation interval values *k*→1, and a decrease was seen from 0.59 to ±0.2. As the distance between the corresponding pixels *(k)* increased, the values of the auto-covariance functions changed only slightly, and *r*→ ± 0.2 at *k* = 5000.

The pixel covariance values for the other plants (kfr2) and agricultural land (kfr3) (Figure 6b,c) also showed a peak of r →1 at the beginning of the quantisation interval (k = 1) (as in the case of the *H. sosnowskyi* plants). However, the remaining covariance value results were smaller than for *H. sosnowskyi* (kfr1), and dropped below 0.10 as the quantisation interval (*k*) increased (Figure 6b,c). Areas with *H. sosnowskyi* plants stand out from the others due to the higher intensity of the pixel brightness.

Based on these calculations, the reliability of the classification result can be assessed because the colour intensity of the plant pixels is not uniform.

#### 3.2.2. Differences in Covariance Values between Plant Classes

Inter-covariance calculations were performed between plant classes, and $K_{\phi }^{\prime }\left(\tau \right)$ was calculated for the following pairs of classes:

- *H. sosnowskyi* plants and other plants (kfr12);
- Other plants and agricultural land (kfr23);
- *H. sosnowskyi* plants and agricultural land (kfr13).

The results are shown in Figure 7.

The correlation between *H. sosnowskyi* plants and other plants (kfr12) varied from ±0.07 to ±0.13 (Figure 7a). The highest value of r = 0.442 was observed for k = 1, with a secondary peak of r = 0.139 at k = 101 and fluctuations of up to 0.1 throughout the interval (Figure 7a).

The highest value of the correlation between other plants and agricultural land (kfr23) was r = 0.937 at k = 1, with a secondary peak of r = 0.304 at k = 101, and then peaks of r = 0.088 at k = 901 and r = 0.044 at k = 4341 (Figure 7b). Overall, the correlation between other plants and agricultural land (kfr23) was ±0.05.

The correlation between *H. sosnowskyi* plants and agricultural land (kfr13) varied in the range ± 0.10. The highest peak was r = 0.345 for k = 1, and secondary peaks were seen at r = 0.066 for k = 901, with fluctuations of up to r = 0.055 throughout the interval (Figure 7c).

The mean covariance values for the pairs of classes are shown in Table 3.

Correlations between *H. sosnowskyi* plants and other plants, and *H. sosnowskyi* plants and agricultural land had covariance values of close to 0.10 (Figure 6 and Figure 7), results that were higher than the correlations between other plants and agriculture land, for which the values did not reach 0.10.

The results for the inter-correlation function for the plant pairs again demonstrate that the geometrical properties of *H. sosnowskyi* are different from those of other plants.

**Figure 8:**
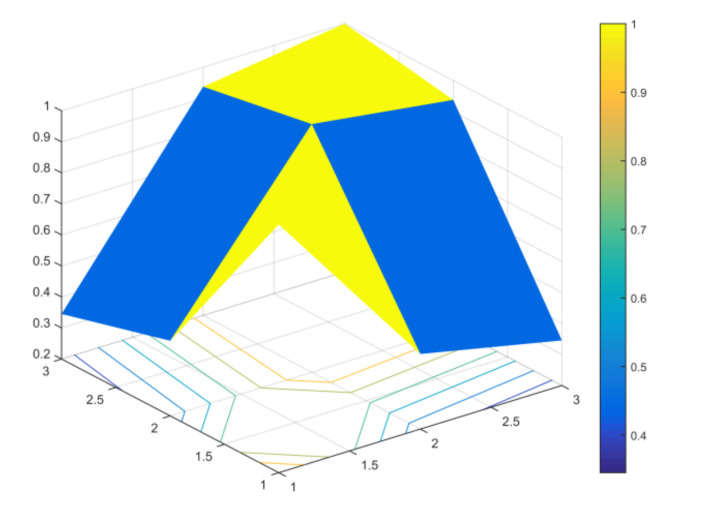
Graphical representation of the (spatial) correlation matrix of the pixel vector arrays for *H. sosnowskyi* plants and other plants.

A generalised (spatial) correlation matrix of the pixel vector arrays for *H. sosnowskyi* and other plants is presented in Figure 8.

The expression of the correlation matrix takes the form of three pyramids (*H. sosnowskyi*, other plants and agricultural land correlation coefficient), in which the values of the correlation coefficients are represented by shades of the colour spectrum. *H. sosnowskyi* is in the yellow colour, other plants and agricultural land–blue colour. The colour projection represented by the horizontal view (x, y array). The x, y value chosen by the algorithm automatically. It is a relative visual expression of the correlation coefficients between different plant classes.

The results for the standard deviation $\sigma_{r}$ were calculated, and based on pixel intensity measurements, the following results were found:

For *H. sosnowskyi*, $\sigma_{pr}\approx$ 50.2; for other plants, $\sigma_{pg}\approx$ 24.5; and for agriculture land, $\sigma_{pb}\approx$ 18.7. The lowest precision was obtained for *H. sosnowskyi* plants (shown in red), and the highest for agricultural land (shown in grey) (Figure 5).

## 4. Discussion and Conclusions

The use of remote sensing in the identification of crops and plants is closely linked to their stage of development [13]. The distinct colours (wavelengths) of visible and near-infrared sunlight reflected by the plants determine the density of green on a patch of land. The period of detection depends on photosynthesis, pigmentation, leaf structure, plant water content, canopy structure, and phenological cycles. Although the values vary with the absorption of red light by plant chlorophyll and the reflection of infrared radiation by water-filled leaf cells, they will be very close to those of other plants in the early vegetation period. Sentinel-2 are optical satellites, and the images produced vary depending on the cloud cover, meaning that it is very important to select an image of an area with no clouds and with atmospheric correction. The high-level data from the satellites were corrected for atmospheric reflectance using cartographic geometry. Heterogeneous terrain significantly complicates signals received by airborne or satellite sensors. It has been demonstrated that both solar direct beam and diffuse skylight illumination conditions are significant factors influencing the anisotropy of reflectance over mountainous areas [29]. However, there are no significant mountains in Lithuania, only relatively small hills and flatlands dominate especially in the centre part of Lithuania. In this study, steep slopes of the surface were not considered. Remotely sensed data are generally analysed assuming flat terrain. As the spectral signature of the ground depends upon the orientation of the surface slope, the topography has to be considered for efficient radiometric corrections in mountainous regions [30].

It was observed that the colour intensity of *H. sosnowskyi* plants in the spring period differed from that of from other plants, and by using an unsupervised classification method it was possible to deduce the mean spectral signatures of these plants. In this study, the estimated value for the mean spectral signature was 956, but this value would be different if images from a different time period and with a different colour intensity were used. Based on this calculated value of the mean spectral signature, supervised automatic classification was performed, which allowed areas of *H. sosnowskyi* plants to be isolated within the selected region. This constituted the data preparation work for the correlation and cross-correlation calculations and studies of isolated plant areas. Our calculations showed that the auto-covariance functions of *H. sosnowskyi* plants, other plants and agricultural land were different. The normalised probability dependence of the pixel vector for *H. sosnowskyi* on the auto-covariance function decreased to r = ± 0.2 as the quantisation interval increased, while the pixel arrays for other plants and agriculture land had values of close to zero. This shows that *H. sosnowskyi* pixels grouped into regions were different from those of other plants, and that the density of the chemical compounds in *H. sosnowskyi* plants was relatively high compared to those of other plants.

A correlation analysis of the different plant groups also showed that the correlation between the pixels of *H. sosnowskyi* plants and other plants was rather weak, possibly because their structures are markedly different. The accuracy of the correlation coefficients calculated here shows that the lowest precision was obtained for *H. sosnowskyi* plants (50.2), and the highest for agriculture land (18.7). To assess the reliability of the classification results, the classification data obtained here should be compared with regional areas recorded as abandoned by the National Paying Agency and with areas recorded as *H. sosnowskyi*.

Traditionally, only optical data have been used to classify types of crops [8], but a combination of optical and synthetic aperture radar (SAR) systems has recently been used in some studies. SAR data have mainly been used to provide complementary information to optical data [31]. The results from optical data have been used as training data to detect changes in the signal observed by an SAR [32]. Change detection can be performed by unsupervised or supervised classification [33]. The main indicator of crop structure from SAR data is the backscattering coefficient (BSC) [34], which represents the average reflectivity of the scene per unit of area and is generally expressed in decibels. The BSC is presented in the form of a histogram, and depending on the data will show one or more peaks of different magnitudes. The backscattering characteristics of several types of crop have been studied in detail in different environments and at varying wavelengths by Harfenmeister et al. (2019) who highlighted the importance of different backscattering parameters, as well as the acquisition time period, in order to detect within-field variability [35]. One of the limitations of working with SAR data is the need for data pre-processing, which may include applying an orbit file, radiometric calibration, de-bursting, multi-looking, speckle filtering, and terrain correction [32]. These processes determine the need for specialist software and time, and can be difficult to implement. Unlike data from optical sensors, the visualisation of SAR data does not provide much useful information about the scene, and it is only after aperture synthesis processing that the polarimetric data are transformed into an interpretable image which is useful when studying the structure of the observed surface and performing unsupervised image classifications. After comparing the classification results and accuracy of optical and SAR data, Stych et al. (2019) observed that a better spatial resolution was evident for optical images [36]. This approach is also useful in small-scale case studies, due to the better spatial resolution with a suitable spectral resolution.

In summary, the classification of plant types can be carried out based on optical data, SAR data, or a combination of both when the changes in the crop area over time needed to be analysed. Optical image classification does not require data pre-processing in the same way as for SAR data, and can be used as training data to detect changes in the signal observed by an SAR. In this paper, we have proposed a method that combines supervised and unsupervised optical data classification with an evaluation of the reliability of the results, based on the covariance function, and have shown that the covariance functions of *H. sosnowskyi* plants, other plants, and agricultural land are different due to the different brightnesses of the pixel colours.

## Figures and Tables

**Table 1:** Results for spectral signatures.

Crop Classes	Colour of Band	Band 2 (blue)	Band 3 (green)	Band 4 (red)	Mean Spectral Signature B3, B4
*H. sosnowskyi*		473	1090	822	956
Forest		356	870	816	843
Pathway		694	1130	1264	1197

**Table 2:** Results for spectral characteristics for pairs of classes.

Crop Classes	Euclidean Distance, Pixels	Spectral Angle, °	Bray–Curtis Similarity, %
*H. sosnowskyi*–forest	682	8	47
*H. sosnowskyi*–pathway	543	11	81
Forest–pathway	1166	12	43

**Table 3:** Mean covariance values for pairs of plant classes.

Plant Classes	Mean Covariance Value (r)
kfr12	±0.10
kfr23	±0.05
kfr13	±0.10

## Data Availability

The data presented in this study are available on request from the corresponding author.
